# Prevalence of TB/HIV Co-Infection in Countries Except China: A Systematic Review and Meta-Analysis

**DOI:** 10.1371/journal.pone.0064915

**Published:** 2013-05-31

**Authors:** Junling Gao, Pinpin Zheng, Hua Fu

**Affiliations:** School of Public Health, Fudan University, Key Laboratory of Public Health Safety, Ministry of Education, Shanghai, China; University of Nebraska Medical Center, United States of America

## Abstract

**Background:**

TB and HIV co-epidemic is a major public health problem in many parts of the world. But the prevalence of TB/HIV co-infection was diversified among countries. Exploring the reasons of the diversity of TB/HIV co-infection is important for public policy, planning and development of collaborative TB/HIV activities. We aimed to summarize the prevalence of TB and HIV co-infection worldwide, using meta-analysis based on systematic review of published articles.

**Methods:**

We searched PubMed, Embase, and Web of Science for studies of the prevalence of TB/HIV co-infection. We also searched bibliographic indices, scanned reference lists, and corresponded with authors. We summarized the estimates using meta-analysis and explored potential sources of heterogeneity in the estimates by metaregression analysis.

**Results:**

We identified 47 eligible studies with a total population of 272,466. Estimates of TB/HIV co-infection prevalence ranged from 2.93% to 72.34%; the random effects pooled prevalence of TB/HIV co-infection was 23.51% (95% CI 20.91–26.11). We noted substantial heterogeneity (Cochran’s *χ* ^2^ = 10945.31, p<0.0001; *I* ^2^ = 99.58%, 95% CI 99.55–99.61). Prevalence of TB/HIV co-infection was 31.25%(95%CI 19.30–43.17) in African countries, 17.21%(95%CI 9.97–24.46) in Asian countries, 20.11%(95%CI 13.82–26.39) in European countries, 25.06%(95%CI 19.28–30.84) in Latin America countries and 14.84%(95%CI 10.44–19.24) in the USA. Prevalence of TB/HIV co-infection was higher in studies in which TB diagnosed by chest radiography and HIV diagnosis based on blood analyses than in those which used other diagnostic methods, and in countries with higher prevalence HIV in the general population than in countries with lower general prevalence.

**Conclusions:**

Our analyses suggest that it is necessary to attach importance to HIV/TB co-infection, especially screening of TB/HIV co-infection using methods with high sensitivity, specificity and predictive values in the countries with high HIV/AIDS prevalence in the general population.

## Introduction

The human immunodeficiency virus (HIV) pandemic presents a significant challenge to global tuberculosis (TB) control [1]. In 2011, 1.1 million (13%) of the 8.7 million people who developed TB worldwide were HIV-positive [2]. TB is a leading killer among people living with HIV. At least one in four deaths among people living with HIV can be attributed to TB [3]. To mitigate the dual burden of TB/HIV in populations at risk of or affected by both diseases, the World Health Organization (WHO) published a document on priority research questions in 2010 [3], and an updated policy on collaborative TB/HIV activities in 2012 [1], which emphasize the importance of surveillance of HIV among TB patients and surveillance of active TB patients among people living with HIV in all countries.

A wide range of estimates for the prevalence of TB/HIV co-infection has been reported. However, to our knowledge, there was only one study, which systematically evaluated the Chinese prevalence data of TB/HIV co-infection, indicated 0.9% (0.6%–1.4%) of TB patients were HIV infected, and the prevalence of TB among HIV/AIDS population was 7.2% (4.2%–12.3%) [4]. So an updated synthesis of the prevalence data from all other countries over the world would be important for public policy and planning and development of clinical services addressing the needs of TB and HIV/AIDS patients. It is also crucial to inform future projects by identifying by identifying the reasons leading to the disparity of TB/HIV co-infection.

We did a systematic review and meta-analysis to establish the prevalence of TB/HIV co-infection. We explored by meta regression the reasons for variations between the primary studies and examined whether prevalence varied by year of publication, sex, study type, surveillance method, study size, study region, HIV prevalence and TB prevalence of general population.

## Methods

### Search Strategy and Selection Criteria

We searched PubMed, Embase and Web of Science with the term “co-infection and (tuberculosis or TB) and (HIV or AIDS or human immunodeficiency virus or acquired immunodeficiency syndrome)” to identify English articles on studies of the prevalence of TB/HIV co-infection until Dec 31, 2011. We also searched relevant reference lists and relevant journals by hand and corresponded with authors. Our analyses accorded with the preferred reporting items for systematic reviews and meta-analyses (PRISMA) guidelines (when appropriate) for a systematic review of prevalence [5].

Cross-sectional or cohort studies addressing the prevalence of HIV infection among TB patients or the prevalence of TB among HIV/AIDS population were included. If the study was reported in duplicate, the article published earlier was included. Investigations were included irrespective of diagnostic methods, but mostly included chest radiography for tuberculosis and blood tests for HIV. Diagnoses based on questionnaires (e.g. self-report of disease status) were also included.

Review articles, and studies in languages other than English or studies conducted in China, with <50 participants, focusing on incidence rates, and addressing specific high risk populations (e.g. injecting drug users or offenders), were excluded.

Two reviewers (JLG and PPZ) independently extracted information about geographical location, year of publication, method of sample selection, sample size, mean age range, surveillance method, diagnostic method, and numbers diagnosed with tuberculosis or HIV from every eligible study. Disagreement was resolved by consensus between the two reviewers or through consultation with the corresponding author, when necessary. If needed, we sought further clarifications from the authors of relevant studies.

### Statistical Analysis

We calculated prevalence estimates with the variance stabilizing double arcsine transformation [6]. Because the inverse variance weight in fixed-effects meta-analyses is suboptimum when dealing with binary data with low prevalence. Additionally, the transformed prevalence is weighted very slightly towards 50%, and studies with prevalence of zero can thus be included in the analysis. We used the Wilson method [7] to calculate 95% CIs around these estimates because the asymptotic method produces intervals which can extend below zero [8]. We estimated heterogeneity between studies with Cochran’s Q (reported as *χ* ^2^ and p values) and the *I* ^2^ statistic, which describes the percentage of variation between studies that is due to heterogeneity rather than chance [9], [10]. Unlike Q, *I* ^2^ does not inherently depend on the number of studies included; values of 25%, 50%, and 75% show low, moderate, and high degrees of heterogeneity, respectively. Because heterogeneity was high (*I* ^2^>75%), we used random-effects models for summary statistics [10]. These models (in which the individual study weight is the sum of the weight used in a fixed-effects model and between-study variability) produce study weights that mainly show between-study variation and thus provide close to equal weighting. We did a sensitivity analyses by excluding one largest study [11].

### Heterogeneity

We further investigated potential sources of heterogeneity by arranging groups of studies according to potentially relevant characteristics and by meta regression analysis, which attempts to relate differences in effect sizes to study characteristics. Factors examined both individually and in multiple-variable models were year of publication, sex (by comparing male samples with female samples), diagnosis method (by comparing studies that diagnosed TB by chest radiography and HIV by blood with others studies), screening method (by comparing studies that screening TB patients among HIV/AIDS population with screening HIV infection among TB patients), place (by comparing population-based studies with hospital-based studies), study type (by comparing cross-sectional studies with cohort studies), study size (as a continuous variable),and estimates of HIV and TB prevalence in the general population of the study country (as a continuous variable). Information about the prevalence of TB and HIV infection in the general population was obtained from the UN Millennium Development Goals Database [12], [13]. We chose the national data that most closely matched the year of publication of the study. We entered only factors that we deemed significant individually (p<0.05) into a multiple regression model to avoid model instability. We did all analyses in Stata (version 12.1) with the commands metan (for random-effects meta-analysis specifying three variables: double-arcsine-transformed prevalence, Wilson CIs, and prevalence ratios) and metareg (for meta regression).

## Results

**Figure 1:**
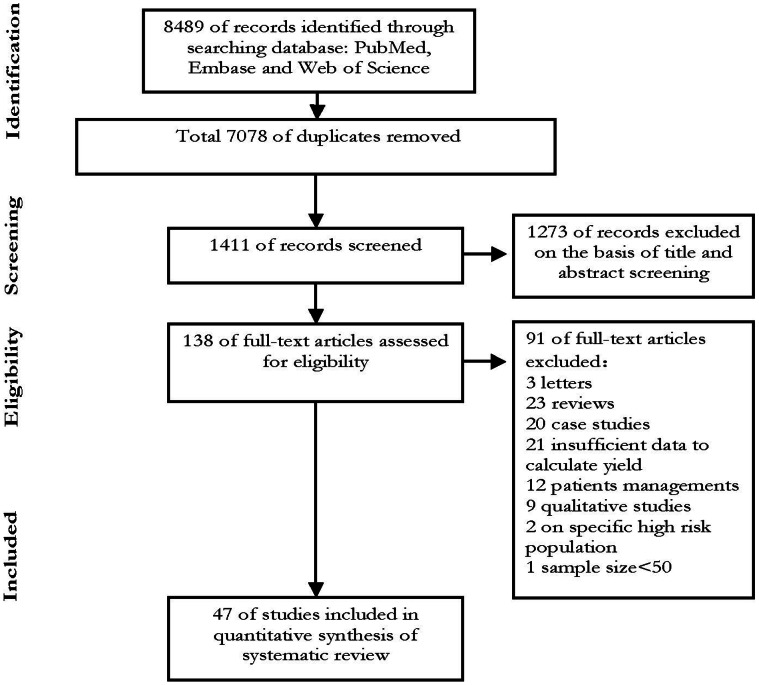
Study flow.

Our searches returned a total of 8489 records. After removal of duplicates and initial screening, we reviewed 138 papers in full. After exclusion of ineligible reports, our final sample was 47 studies (n = 272,466) were included in this review [11], [14]–[59] (**Figure 1**). The characteristics of the included studies were shown in **Table S1**.

Six reports each were from the USA (226,904)[11], [17]–[19], [22], [54] and Brazil (4,841) [29], [37], [42], [46], [49], [56], five were from Nigeria (2,520) [21], [32], [47], [52], [55], four each were from Ethiopia (2,609) [28], [34], [36], [44] and Spain (10,809) [14], [24], [26], [40], three were from India (1,825) [27], [45], [59], two each were from Iran (721) [43], [53], South Africa (374) [23], [50], Zambia (2,309) [16], [35], and Zimbabwe (2,049) [15], [58], and one each was from Cambodia (441) [25], Trinidad and Tobago (121) [41], Israel (1,059) [51], Netherland (13,269) [30], Singapore (184) [48], Tanzania (233) [39], Thailand(54) [31], Togo(569) [57], UK(157) [20], Ukraine(968) [33], and Vietnam(450) [38]. Twenty-nine reports screened HIV infection among TB patients (n = 22,884)[15]–[17], [19]–[23], [26]–[28], [31], [33]–[36], [38], [41], [42], [48], [50]–[52], [54]–[59], and eleven reports screened TB patients among HIV/AIDS patients (12,627)[14], [25], [32], [39], [40], [43]–[47], [53], and the screening method was unknown in the remaining seven (236,955) [11], [18], [24], [29], [30], [37], [49]. Tuberculosis was diagnosed by combination of chest radiography and sputum culture in 9 studies (n = 6,562) [15], [28], [31], [34], [36], [39], [42], [50], [58], by combination of chest radiography, sputum culture and PPD skin test in 1 studies (459) [43], by sputum culture in 17 studies (14,769)[14], [16], [17], [22], [25]–[27], [32], [33], [38], [41], [44], [45], [47], [48], [52], [54], by PPD skin test in 1 study (262) [53], by chest radiography in 1 study (2072) [35], by combination of chest radiography and PPD skin test in 1 studies (161) [23]; the method of diagnosis was unknown in the remaining seventeen(248,181)[11], [18]–[21], [24], [29], [30], [37], [40], [46], [49], [51], [55]–[57], [59]. HIV/AIDS was diagnosed by blood in 25 studies (14,953)[15]–[17], [20], [21], [23], [27], [28], [33]–[36], [39], [41], [43], [44], [47], [48], [51], [52], [55]–[59], the method of diagnosis was unknown in the remaining twenty-two studies (256,913) [11], [14], [18], [19], [22], [24]–[26], [29]–[32], [37], [38], [40], [42], [45], [46], [49], [50], [53], [54].

For study types, four were prospective cohort studies (n = 9,561) [20], [28], [40], [58], nine studies were retrospective cohort studies (242,934)[11], [18], [22], [30], [42], [49]–[51], [54], and the remaining thirty four studies were cross sectional studies (19,971)[14]–[17], [19], [21], [23]–[27], [29], [31]–[39], [41], [43]–[48], [52], [53], [55]–[57], [59], fifteen studies were population-based (255,093)[11], [18], [24], [26], [29], [30], [36]–[40], [42], [49], [54], [58], and the other 32 studies were hospital-based (17,373)[14]–[17], [19]–[23], [25], [27], [28], [31]–[35], [41], [43]–[48], [50]–[53], [55]–[57], [59], twenty one reported data for men and women respectively (men = 13,004 women = 23,811) [15], [17], [18], [20], [28], [30], [33], [36], [38], [39], [44], [46]–[48], [52]–[55], [57]–[59].

**Figure 2:**
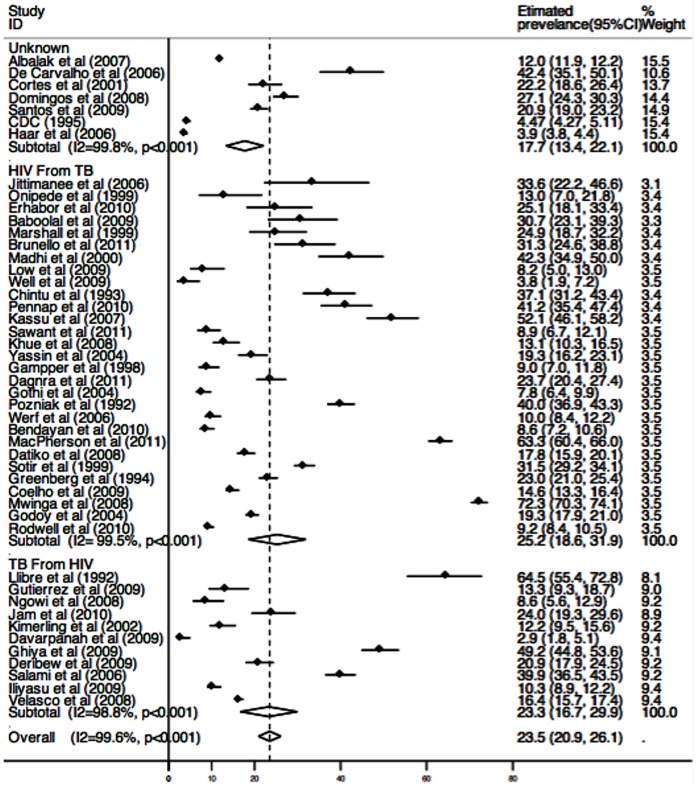
Estimated prevalence of TB HIV co-infection by Screen methods.

**Figure 3:**
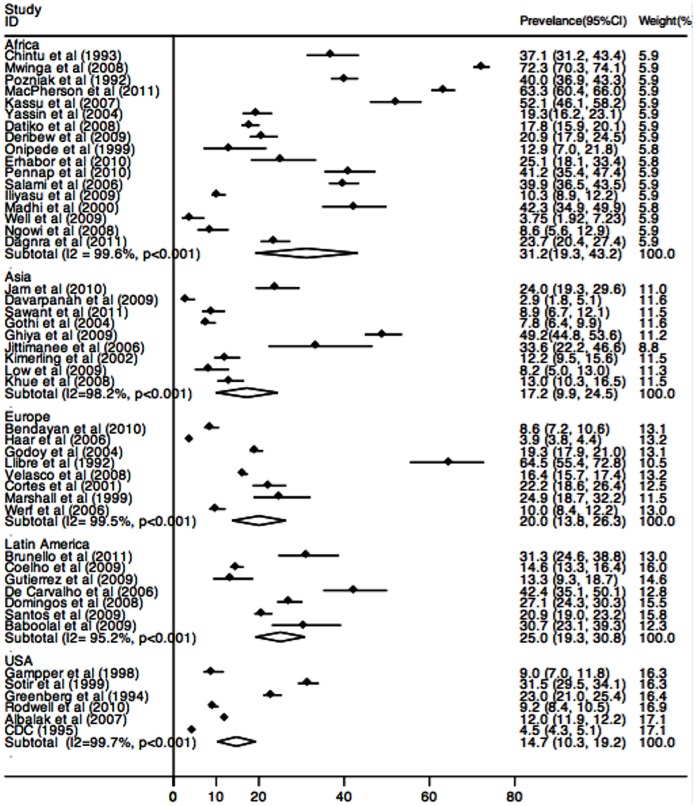
Estimated prevalence of TB and HIV co-infection by Region.

Estimates of TB/HIV co-infection prevalence ranged from 2.93% to 72.34% (**Figure 2**); heterogeneity was substantial (*χ* ^2^ = 10945.31, p<0.0001; *I* ^2^ = 99.58%, 95% CI 99.55–99.61). The random effects pooled prevalence of TB/HIV co-infection was 23.51% (95% CI 20.91–26.11). The random effects pooled TB/HIV co-infection prevalence among TB patients was 25.23%(95%CI 18.58–31.89) with substantial heterogeneity (*χ* ^2^ = 5478.67, p<0.0001; *I* ^2^ = 99.49%, 95% CI 99.43–99.54). And the random effects pooled TB/HIV co-infection prevalence among HIV/AIDS patients was 23.30%(95%CI 16.69–29.91) with substantial heterogeneity (*χ* ^2^ = 850.64, p<0.0001; *I* ^2^ = 98.82%, 95% CI 98.51–99.07). Prevalence of TB/HIV co-infection was 31.25%(95%CI 19.30–43.17) in African countries, 17.21%(95%CI 9.97–24.46) in Asian countries, 20.11%(95%CI 13.82–26.39) in European countries, 25.06%(95%CI 19.28–30.84) in Latin America countries and 14.84%(95%CI 10.44–19.24) in the USA (**Figure 3**).

As part of our sensitivity analyses, we excluded one large tuberculosis study [11]; estimates of TB/HIV co-infection prevalence did not change. Whereas the random-effects pooled TB/HIV co-infection prevalence raised to 23.89% (95% CI 20.21–27.56) with substantial heterogeneity (*χ* ^2^ = 10401.68, p<0.0001; *I* ^2^ = 99.57%, 95% CI 99.53–99.60).

Meta regression analyses were used in order to explore source of heterogeneity. In individual variable meta regression analysis, the prevalence of TB/HIV co-infection was higher in studies in which chest radiography was used for TB diagnosis and HIV diagnosis based on blood analyses (coefficient = 13.70, p = 0.001) than in those in which other diagnostic methods were used; general population prevalence of TB (coefficient = 0.03, p = 0.001) and HIV (coefficient = 1.76, p<0.001) were positively related to the prevalence of TB/HIV co-infection, but high general population prevalence of TB was not related to the prevalence of TB/HIV co-infection after multivariate meta regression(**Table 1**).

**Table 1:** Univariate and multivariate metaregression for prevalence of TB.

	Metaregression coefficient (%)	95%CI	P
**Univariate metaregression**			
Year of publication	−0.27	−0.88–0.34	0.381
Sex (male vs. female)	1.35	−8.10–10.80	0.774
Diagnosis (TB by chest radiography and HIV by blood vs. other)	13.70	5.89–21.52	0.001
Screen (HIV from TB vs. TB from HIV)	−4.34	−12.65–3.97	0.302
Place (population-based vs. hospital-based)	−5.24	−12.45–1.97	0.152
Study type (cross-sectional vs. cohort)	1.43	−3.61–6.46	0.575
Sample size (≥500 vs. <500)	−0.21	−7.09–6.67	0.952
Population prevalence of TB	0.03	0.01–0.05	0.001
Population prevalence of HIV/AIDS	1.76	1.14–2.37	<0.001
**Multivariate metaregression**			
Diagnosis (TB by chest radiography and HIV by blood vs. other)	3.32	2.46–12.10	0.035
Population prevalence of TB (per 100 000)	−0.001	−0.03–0.02	0.923
Population prevalence of HIV/AIDS (%)	1.62	0.71–2.53	0.001

## Discussion

Our systematic review and meta-analysis of TB/HIV co-infection identified 47 studies of 272,466 individuals except China. Our main findings were that, a high prevalence of TB/HIV co-infection; TB/HIV co-infection prevalence was higher among TB patients than among HIV/AIDS patients, but which were not significantly different. Additionally, high general population prevalence of HIV/AIDS was related to higher prevalence of TB/HIV co-infection. These findings suggest that it should be paid more attention to screening TB/HIV co-infection among TB patients and HIV/AIDS patients, especially in countries with high HIV prevalence or TB prevalence.

Tuberculosis and HIV/AIDS can be worsened by each other. Tuberculosis is the most common opportunistic disease and cause of the death for those infected with HIV [60]. Similarly, HIV infection is one of the most important risk factors associated with an increased risk of latent TB infection progressing to active TB disease [61], [62]. So the WHO’s Policy on collaborative TB/HIV activities recommends a combination of measures to reduce the burden of TB among HIV-infected individuals [1]. These measures include intensified case finding, isoniazid preventive therapy, and infection control and antiretroviral therapy. The prevalence of TB/HIV co-infection in our meta-analysis is 23.51% (95% CI 20.91–26.11), which is higher than the rate (13%) reported by WHO in 2011 [2]. The reasons may be that, more than half studies (26 of 47 studies) were conducted in African and Asian countries (Ethiopia, Nigeria, South Africa, Zambia, Zimbabwe, India and Thailand) with high general population prevalence of HIV and TB, while the HIV prevalence among people with TB is as high as 80% in some African countries [63]. However, this finding suggests case finding should be intensified among TB patients and HIV/AIDS patients, because only 40% of notified TB cases had a documented HIV test result in 2011 [2], the percentage of people with TB who received an HIV test was just 26% in 2009 [63].

Our heterogeneity analyses generated several potentially important finding. Firstly, diagnostic method of chest radiography and blood was associated with significantly higher prevalence of TB/HIV co-infection than were other diagnostic methods. This finding might be because of the higher sensitivity and specificity of chest radiography compared with sputum culture [64], which suggesting optimal TB algorithm to diagnose TB with high sensitivity, specificity and predictive values, that can be applied across different settings and in patients with different TB and HIV disease burdens should be considered in the future. Secondly, prevalence of TB/HIV co-infection was positively associated with prevalence of HIV in the general population, which is consistent with a previous study result that the rate of TB/HIV co-infection depends on the prevalence of HIV infection in a community [36]. This result is potentially important from a public health perspective because it suggests it is should be paid more attention to screening TB/HIV co-infection among HIV/AIDS patients, especially in countries with high HIV/AIDS prevalence of general population. This is also important from a preventive therapy because preventive TB therapy (any anti-TB drugs) reduces the risk of active TB by 32% in people living with HIV [65], and antiretroviral therapy reduces the individual risk of TB by 54% to 92% and the population-based risk by 27% to 80% among people living with HIV [1]. So HIV-infected patients should receive antiretroviral therapy with optimal combination of antiretroviral and anti-TB therapy as early as possible. The substantial heterogeneity suggests that caution is necessary when pooled estimates are used and emphasizes the need for careful description of samples and diagnostic methods in surveys. Our sensitivity analyses showed that our overall results were not materially different when we excluded one study with largest sample size. Other characteristics that we did not test might have been associated with heterogeneity, such as history of TB and HIV/AIDS, infectious routes of HIV or stages of AIDS, and future research should describe samples in further detail.

Previous meta analysis of TB/HIV co-infection in China [4] reported a lower prevalence than that in our review, this may be because of low prevalence of HIV in China compared with that in countries where studies in our review were conducted. In addition, Gao and colleagues [4] reported lower summarized prevalence of HIV among TB patients for population-based studies. But our review didn’t find relationship between study place and prevalence of TB/HIV co-infection. An explanation could be demographic factors of the study population, such as the gender and age distribution of subjects according to disease severity [66]–[68], but would need verification in future studies.

Because a meta-analysis has synthesized prevalence data of TB/HIV co-infection in China [4],_ENREF_67 so we searched prevalence data of TB/HIV co-infection in all other countries. But only English articles were reviewed in our study, data published in other languages articles (e.g. French, Japanese) was unobtainable. So collaboration of researchers from different language backgrounds is needed in the future. Second, due to the specific high-risk behaviors for TB and HIV infection, the selection of subjects might make results prone to potential selection bias even as we have excluded two studies performed among prisoners and injecting drug users. Another limitation of our systematic review is that, not all-necessary information, even age and HIV infection routes stages of AIDS of the study population, could be obtained from all included studies. Therefore, relevant stratified analyses could not be performed to disclose more detailed characteristics of the co-infection and its related risk factors.

In conclusion, our analyses suggest that it is necessary to attach importance to HIV/TB co-infection, especially screening of TB/HIV co-infection using methods with high sensitivity, specificity and predictive values in the countries with high HIV/AIDS prevalence of general population. As prevalence of TB/HIV co-infection was positively associated with prevalence of HIV in the general population, and antiretroviral therapy is much helpful for treatment of both diseases, so HIV-infected patients should receive antiretroviral therapy with as early as possible.

## Supporting Information

Table S1 **Studies of Prevalence of TB and HIV co-infection.**
(DOC)
