# Supplementary material for: Prevalence of TB/HIV Co-Infection in Countries Except China: A Systematic Review and Meta-Analysis
Source: PLoS One. 2013 May 31;8(5):e64915. doi: 10.1371/journal.pone.0064915 (PMC3669088; doi:10.1371/journal.pone.0064915)
Supplement: Table S1 — Studies of Prevalence of TB and HIV co-infection. (DOC) [file pone.0064915.s001.doc]

**Table S1** Studies of Prevalence of TB and HIV co-infection

| Study | Sampling method | Study type | Age | Screen methods | Diagnostic method | Sample size |
| --- | --- | --- | --- | --- | --- | --- |
| Brunello et al,^56^ 2011 Brazil | New TB cases reported in Ribeirão Preto in 2006 | HB cross sectional study |  | HIV from TB patients | TB: Unknown, HIV:Blood | 160 |
| De Carvalho et al,^29^ 2006 Brazil | Tuberculosis cases occurring in Taubaté in 2001 and 2002 | PB cross sectional study |  | Unknown | Unknow | 165 |
| Gutierrez et al,^46^ 2009 Brazil | A random sample of patients with HIV/AIDS was selected at a reference center for HIV/AIDS care in São Paulo between 4 April 2005 and 5 August 2005 | HB cross sectional study | 41.6 | TB from HIV patients | TB: tuberculin test,  HIV: Unknown | 203 |
| Domingos et al,^37^2008 Brazil | All TB patients reported from January 1, 1996, to December 31, 2000, and residing in Recife were selected | PB cross sectional study |  | Unknown | Unknown | 861 |
| Santos et al,^49^ 2009 Brazil | New TB cases co-infected by HIV, residents in São José do Rio Preto,SP, Brazil, who began treatment between Jan 1, and Dec 31, 2006. | PB retrospective cohort study | 36.3 | Unknown | Unknown | 1457 |
| Coelho et al,^42^ 2009 Brazil | the TB surveillance database in the city of Santos between 2000 and 2004 | PB retrospective cohort study |  | HIV from TB patients | TB:CR and sputum, HIV:Unknown | 1995 |
| Kimerling et al,^25^ 2002 Cambodia | All HIV-positive persons in the Home Care Network in Phnom Penh from 1 September  to 21 October 2000 | HB cross sectional study |  | TB from HIV patients | TB: sputum, HIV:Unknown | 441 |
| Baboolal et al,^41^ 2009 Caribbean | all of the culture-positive Mycobacterium tuberculosis cases in Trinidad and Tobago from October 2006 to September 2007 | HB cross sectional study | 42.8 | HIV from TB patients | TB: sputum, HIV:blood | 121 |
| Kassu et al,^34^ 2007 Ethiopia | All adult TB patients in a teaching hospital in Northwest Ethiopia | HB cross sectional study |  | HIV from TB patients | TB:CR and sputum, HIV:Blood | 257 |
| Yassin et al,^28^ 2004 Ethiopia | TB Patients treated in 5 rural hospitals of the Southern Region of Ethiopia from Sep to Nov 2002 | HB prospective Cohort study | 28 | HIV from TB patients | TB:CR and sputum, HIV:Blood | 500 |
| Deribew et al,^44^ 2009 Ethiopia | All new TB patients in three hospitals in Oromiya regional state of Ethiopia from Feb to Apr, 2009 | HB cross sectional study | 33.4 | TB from HIV patients | TB: sputum, HIV:blood | 591 |
| Datiko et al,^36^ 2008 Ethiopia | All TB patients in the Southern Nations, Nationalities and Peoples' Region (SNNPR) of Ethiopia were consecutively enrolled from Sep, 2004 to Apr,2005. | PB cross-sectional study | 28.4 | HIV from TB patients | TB:CR and sputum, HIV:blood | 1261 |
| Sawant et al,^59^ 2011 India | TB patients at the B.Y.L. Nair Charitable Hospital, Mumbai over a period of 1 year | HB cross sectional study |  | HIV from TB patients | TB: Unknown, HIV:Blood | 432 |
| Ghiya et al,^45^ 2009 India | HIV-positive cases in the HIV Referral Clinic, Department of Skin and Venereal Diseases, Shri Sayajirao General Hospital and Medical College, Vadodara, Gujarat, India, from Jul 2005 to Nov 2007. | HB cross sectional study |  | TB from HIV patients | TB: sputum, HIV:Unknown | 500 |
| Gothi et al,^27^ 2004 India | TB patients in the tuberculosis referral unit of a tertiary care hospital in Mumbai, India between Nov 2000 and Sep 2002, | HB cross sectional study | 42 | HIV from TB patients | TB: sputum, HIV:blood | 893 |
| Jam et al,^53^ 2010 Iran | HIV-seropositive patients in three HIV/AIDS health care centers of Tehran, Iran From Jan 2006 to Feb 2007 | HB cross sectional study | 30 | TB from HIV patients | TB: PPD, HIV:Unknown | 262 |
| Davarpanah et al,^43^ 2009 Iran | HIV-positive individuals who referred to Center for Counseling and Behavioral Modification in Shiraz, Southern Iran were enrolled from Jan 2004 to Dec 2006 | HB cross sectional study |  | TB from HIV patients | TB:CR,PPD and sputum, HIV:blood | 459 |
| Bendayan et al,^51^ 2010 Israel | TB patients hospitalized in the Pulmonary and Tuberculosis Department of Shmuel Harofeh Hospital during the period Jan 2000 to Dec 2006. | HB retrospective cohort study | 34 | HIV from TB patients | TB: Unknown, HIV:Blood | 1059 |
| Haar et al,^30^ 2006 Netherlands | Data were obtained from the national surveillance register of all patients notified with TB from 1993 through 2001 | PB retrospective cohort study |  | Unknown | Unknown | 13269 |
| Onipede et al,^20^ 1999 Nigeria | TB patients treated in a Nigerian chest clinic | HB cross sectional study |  | HIV from TB patients | TB: Unknown, HIV:Blood | 79 |
| Erhabor et al,^52^ 2010 Nigeria | All TB patients treated in the Niger hospital chest clinic were consecutively recruited between Jul and Aug 2006 | HB cross sectional study | 39.5 | HIV from TB patients | TB: sputum, HIV:blood | 120 |
| Pennap et al,^55^ 2010 Nigeria | All TB patients treated in a rural tuberculosis referral clinic in northern Nigeria between Mar 2007 and Aug 2008 | HB cross sectional study |  | HIV from TB patients | TB: Unknown, HIV:Blood | 257 |
| Salami et al,^32^ 2006 Nigeria | HIV infected patients in the University of Ilorin teaching hospital in Nigeria between Jan 2000 and Dec 2004. | HB cross sectional study |  | TB from HIV patients | TB: sputum, HIV:Unknown | 744 |
| Iliyasu et al,^47^ 2009 Nigeria | HIV/AIDS patients attending the  Aminu Kano Teaching Hospital HIV/AIDS specialist clinic from Jan to Dec 2006. | HB cross sectional study | 34.2 | TB from HIV patients | TB: sputum, HIV:blood | 1320 |
| Low et al,^48^ 2009 Singapore | All TB patients treated at the  Singapore General Hospital between Sep 2005 and Dec 2006 | HB cross sectional study |  | HIV from TB patients | TB: sputum, HIV:blood | 184 |
| Madhi et al,^23^ 2000 South Africa | All children TB patients treated at the three hospitals affiliated with the Department of Paediatrics at the University of the Witwatersrand, Johannesburg, South Africa between Aug 1996 and Jan 1997 | HB cross sectional study | 2 ms to 12 ys | HIV from TB patients | TB:CR,PPD HIV:Blood | 161 |
| Well et al,^50^ 2009 South Africa | all children TB patients at Tygerberg Hospital in Cape Town, South Africa between Jan 1985 and Apr 2005 | HB retrospective cohort study | 28 month | HIV from TB patients | TB:CR ,sputum  HIV:Unknown | 213 |
| Llibre et al,^14^ 1992 Spain | HIV-infected patients admitted to Sant Jaume Hospital in Calella Spain From Apr 1984 to Dec 1989 | HB cross sectional study | 28.4 | TB from HIV patients | TB: sputum, HIV:Unknown | 113 |
| Mayoral et al,^24^ 2001 Spain | TB patients admitted to 4 public hospital in Seville Spain in 1998 | PB cross sectional study |  | Unknown | Unknown | 427 |
| Godoy et al,^26^ 2004 Spain | New cases of tuberculosis in residents of Catalonia, Spain from May 1996 to April 1997 | Population-based cross sectional study |  | HIV from TB patients | TB: sputum, HIV:Unknown | 2508 |
| Velasco et al,^40^ 2008 Spain | All TB patients in residents of Madrid, Spain between 1987 and 2004 | PB Prospective Cohort study | 31.7 | TB from HIV patients | Unknown | 7761 |
| Ngowi et al,^39^ 2008 Tanzania | HIV patients at Haydom Lutheran Hospital in northern rural Tanzania from Sep 2006 to Mar 2007. | HB cross sectional study | 35.5 | TB from HIV patients | TB:CR, sputum, HIV:Blood | 233 |
| Jittimanee et al,^31^ 2006 Thailand | TB patients admitted to a hospital in Bangkok,Thailand | HB cross sectional study |  | HIV from TB patients | TB:CR, sputum HIV:Unknown | 54 |
| Dagnra et al,^57^ 2011 Togo | TB patients in four diagnosis and treatment centers in Togo in Dec 2007 | HB cross-sectional study |  | HIV from TB patients | TB: Unknown, HIV:Blood | 569 |
| Marshall et al,^20^ 1999 UK | 157 consecutive patients in an inner London hospital From Nov 1996 to Oct 1997. | HB Prospective Cohort study |  | HIV from TB patients | TB: Unknown, HIV:Blood | 157 |
| Werf et al,^33^ 2006 Ukraine | All TB patients who were >18 years of age and living in Kiev City, Ukraine from Mar 2004 to Feb 2005 | HB cross sectional study |  | HIV from TB patients | TB: sputum, HIV:blood | 968 |
| Gampper et al,^19^ 1998 USA | HIV patients treated at 4 HIV clinics in Rhode Island from Jan 1992 to Dec 1994 | HB cross sectional study |  | TB from HIV patients | Unknown | 549 |
| Sotir et al,^22^ 1999 USA | All patients with tuberculosis disease diagnosed at Grady Memorial Hospital between Jan 1991 and Dec 1997 | HB retrospective cohort study | 40.5 | HIV from TB patients | TB: CR ,sputum, HIV:blood | 1378 |
| Greenberg et al,^17^ 1994 USA | TB patients attending nine chest clinics (1989-1991) and one inner-city hospital (1990-1991) in New York City, USA | HB cross sectional study |  | HIV from TB patients | TB: CR ,sputum, HIV:blood | 2270 |
| Rodwell et al,^54^ 2010 USA | Routine TB surveillance data in San Diego County from 1993 through 2007 | PB retrospective cohort study |  | HIV from TB patients | TB: CR ,sputum, HIV:Unknown | 2787 |
| CDC,^18^ 1995 USA | TB patients(1989-1993) and AIDS patients(1989-Mar 1994) registered in the Chicago Department of Public Health | PB retrospective cohort study |  | Cross screening | Unknown | 9798 |
| Albalak et al,^11^ 2007 USA | TB cases reported to the Centers for Disease Control and Prevention national TB surveillance system from all 50 states and the District of Columbia from 1993 through 2004. | PB retrospective cohort study |  | Unknown | Unknown | 210978 |
| Khue et al,^38^ 2008 Vietnam | All TB patients living in Haiphong City, Vietnam from Jun to Dec 2006 | PB cross sectional study | 45 | HIV from TB patients | TB: sputum, HIV:blood | 450 |
| Chintu et al,^16^ 1993 Zambia | All newly diagnosed cases of TB in children seen at the Paediatric Department of the University Teaching Hospital in Lusaka,Zambia, between April 1,1990 and October 31,1991 | HB cross sectional study | ≤14 | HIV from TB patients | TB: sputum, HIV:blood | 237 |
| Mwinga et al,^35^ 2008 Zambia | TB patients treated at three clinics in the Livingstone District in Southern Province of Zambia from Sep 2004 to Dec 2006 | HB cross sectional study |  | HIV from TB patients | TB:CR, HIV:Blood | 2072 |
| Pozniak et al,^15^ 1992 Zimbabwe | TB patients admitted to the City of Harare tuberculosis unit between Jun 1988 to Sep 1989 | HB cross sectional study | 33.4 | HIV from TB patients | TB:CR, sputum, HIV:Blood | 906 |
| MacPherson et al,^58^ 2011 Zimbabwe | Participants came from 46 neighborhood clusters in Harare were recruited between February 2006 and July 2007 | PB Cohort study | 37.1 | HIV from TB patients | TB:CR, sputum, HIV:Blood | 1143 |

BP: Population-based, HB: Hospital-based, CR: Chest radiography
